# Adverse events after catheter ablation of atrial fibrillation: a comprehensive nationwide cohort study

**DOI:** 10.1007/s10840-026-02328-4

**Published:** 2026-04-21

**Authors:** Antti Lappalainen, Juha E. K. Hartikainen, Konsta Teppo, Olli Halminen, Aapo L. Aro, Jarkko Karvonen, Rasmus Siponen, Annukka Marjamaa, Birgitta Salmela, Jukka Putaala, Pirjo Mustonen, Miika Linna, Jari Haukka, K. E. Juhani Airaksinen, Mika Lehto

**Affiliations:** 1 Heart Center, Kuopio University Hospital and University of Eastern Finland, Kuopio, Finland; 2 Department of Internal Medicine, University of Turku, Turku, Finland; 3 Cardiac Unit, Satasairaala, Pori, Finland; 4 Department of Health and Social Management, University of Eastern Finland, Kuopio, Finland; 5 Heart and Lung Center, Helsinki University Hospital and University of Helsinki, Helsinki, Finland; 6 University of Eastern Finland, Kuopio, Finland; 7 Department of Internal Medicine, Heart Center, Päijät-Häme Central Hospital, Lahti, Finland; 8 Department of Neurology, Helsinki University Hospital and University of Helsinki, Helsinki, Finland; 9 Heart Centre, Turku University Hospital and University of Turku, Turku, Finland; 10 Department of Industrial Engineering and Management, Aalto University, Espoo, Finland; 11 University of Helsinki, Helsinki, Finland; 12 Department of Internal Medicine, Jorvi Hospital, HUS Helsinki University Hospital and University of Helsinki, Helsinki, Finland

**Keywords:** Atrial fibrillation, Catheter ablation, Complications, Adverse events

## Abstract

**Background:**

Catheter ablation is frequently used for rhythm control of atrial fibrillation (AF). However, nationwide, real-world data on the incidence of adverse events and their predictors following the procedure remain limited.

**Methods:**

Finnish AntiCoagulation in Atrial Fibrillation (FinACAF) is a registry-linkage study including all patients with AF in Finland between 2007 and 2018. We investigated the incidence and predictors of new-onset adverse events following all first-time AF catheter ablations (3075 patients) in Finland between 2012 and 2016.

**Results:**

At the one-month follow-up, 1.14% and by two years 6.34% of the patients experienced an adverse event or died. At one-month follow-up, 0.33% of patients developed ischaemic stroke or transient ischaemic attack (IS/TIA). The incidence of IS/TIA was 6.6 times higher during the first 19 days after ablation compared to the subsequent follow-up period. The incidence of IS/TIA after anticoagulation discontinuation was low: 0.8 events per 100 patient-years. The most frequent events during two-year follow-up were bleeding (2.44%), IS/TIA (1.82%), and heart failure (1.24%). Older age was associated with all-cause mortality, bleeding, heart failure and combined endpoint of adverse events during two-year follow-up. Women presented with a higher risk of IS at the time of ablation, but the sex-related difference attenuated during the follow-up. Additionally, the CHA₂DS₂-VA score predicted IS, but not the combined endpoint of IS/TIA or TIA alone, during the follow-up period.

**Conclusion:**

In this nationwide, real-world cohort, older age, especially ≥ 70 years, emerged as the strongest risk factor for adverse events after first-time catheter ablation for AF.

**Graphical Abstract:**

**Figure:**
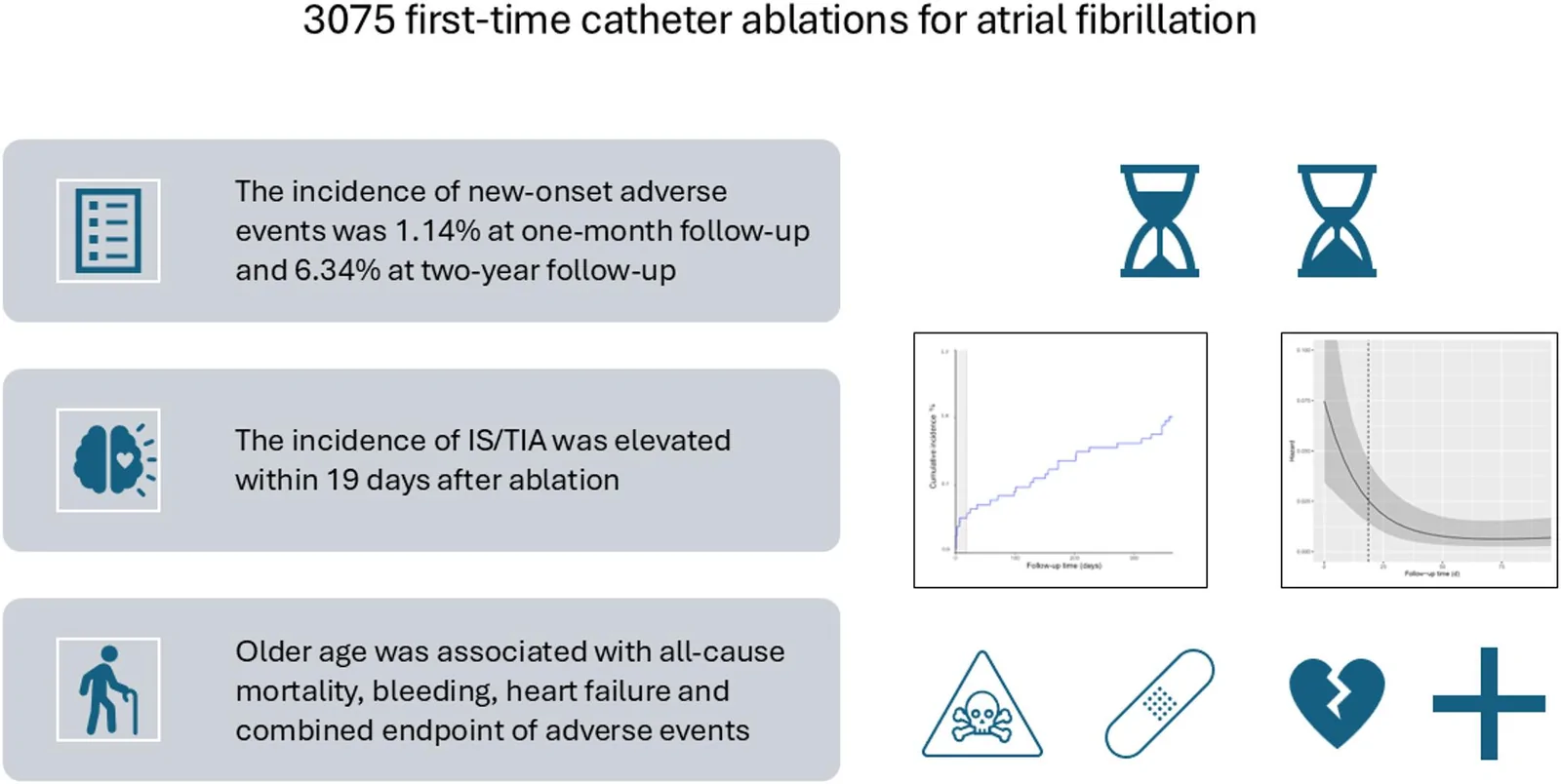

**Supplementary Information:**

The online version contains supplementary material available at https://doi.org/10.1007/s10840-026-02328-4.

## Introduction

Atrial fibrillation (AF) is the most common cardiac arrhythmia, with a globally increasing prevalence driven by an aging population, a growing burden of comorbidities, and advancements in detection technologies [1–4]. Catheter ablation is a well-established and increasingly used treatment to prevent AF recurrences and to relieve symptoms [1, 5–7]. According to current guidelines, catheter ablation is the recommended treatment for rhythm control in symptomatic patients with AF who do not tolerate or respond to antiarrhythmic drugs. Catheter ablation may also be considered a first-line treatment in selected patients, such as those with heart failure and reduced left ventricular ejection fraction due to inadequate AF rate control, given its potential prognostic benefits [1, 8, 9].

Catheter ablation is also increasingly used in elderly patients [7]. However, somewhat contradictory results regarding outcomes and complications in this patient group have been reported [10]. The Danish nationwide cohort study found no statistically significant difference in periprocedural complications between patients aged ≥ 75 years and those aged 65–74 years [11]. Correspondingly, the randomized CABANA trial (Catheter Ablation vs. Antiarrhythmic Drug Therapy for Atrial Fibrillation) found no differences by age in treatment-related complications during long-term follow-up [12]. On the contrary, several meta-analyses have reported that elderly patients have higher risk of peri- and post-procedural complications compared to their younger counterparts [13–15].

Many large-scale studies and meta-analyses suggest that women have a higher risk of complications following AF ablation [16–18]. In contrast, a large multicentre retrospective cohort study reported that, after multivariable adjustment, sex was not significantly associated with complications, suggesting that the observed differences may be attributable to baseline characteristics and procedural factors rather than sex alone [19].

Most evidence on the success and adverse events of catheter ablation for AF comes from selective patient populations in optimal conditions; randomized clinical trials, studies conducted at high-volume centres, and meta-analyses pooling such data [9, 10, 13, 20, 21]. To gain a comprehensive picture of catheter ablation–related complications, nationwide real-world data on patients undergoing catheter ablation for AF, covering all levels of the healthcare system, are needed. Therefore, we conducted a nationwide cohort study including all patients in Finland who underwent their first-time AF catheter ablation between 2012 and 2016. We examined the incidence of new-onset adverse events during one-month and two-year follow-up periods after catheter ablation, and studied whether clinical characteristics such as age, sex, and comorbidities were associated with increased risk of adverse outcomes.

## Methods

### Study population

**Fig. 1:**
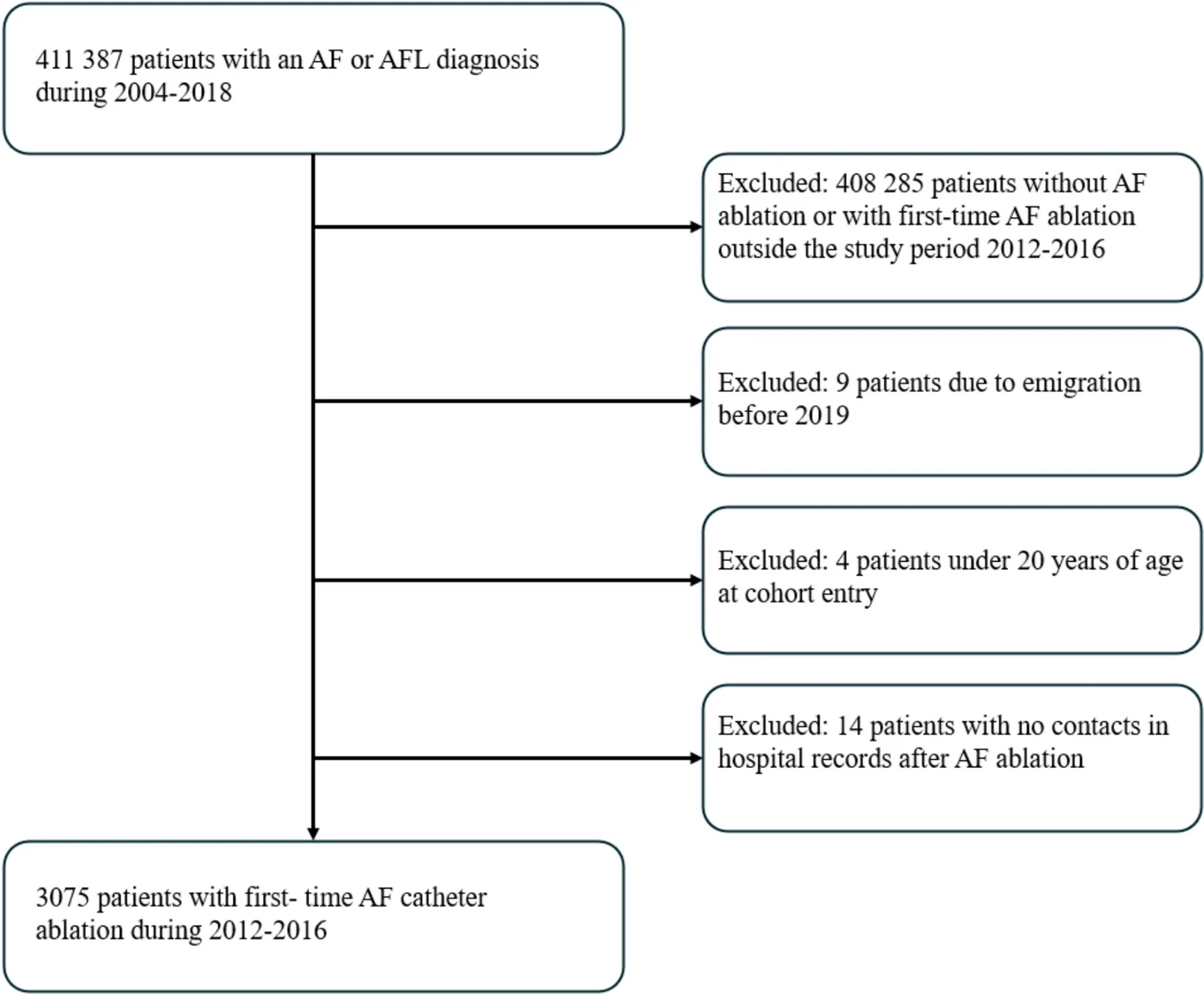
Flowchart of the patient selection process

The Finnish AntiCoagulation in Atrial Fibrillation (FinACAF) Study (ClinicalTrials Identifier: NCT04645537; ENCePP Identifier: EUPAS29845) is a nationwide retrospective cohort study including all patients documented with AF and atrial flutter (AFL) in Finland from 2004 to 2018 encompassing a total of 411 387 patients [22]. Patients were identified using all national healthcare registers, including hospitalizations and outpatient specialist visits (HILMO), primary healthcare (AvoHILMO), drug prescriptions (the National Reimbursement Register maintained by the Social Insurance Institute, KELA) and deaths (Statistics Finland). The cohort inclusion criterion was an International Classification of Diseases, Tenth Revision (ICD-10) diagnosis code of I48, encompassing AF and AFL. The Nordic Classification of Surgical Procedures (NCSP) codes identifying AF catheter ablations were available in Finland from the registries since 2011. Therefore, we started the evaluation of procedures from 2012 and excluded the year 2011 as a transition year. In this sub-study we excluded patients under 20 years old at the time of first AF diagnosis, those emigrating before the end of 2018, and those who did not have any further recordings in the hospital registries after the ablation (Fig. 1). We searched for all patients who underwent their first-time AF catheter ablation (NCSP procedural code TFP46) between 2012 and 2016, allowing a two-year follow-up period after the ablation. Baseline clinical characteristics of the patients were defined the day before the catheter ablation. Moreover, we assessed the proportion of patients who had filled prescriptions for reimbursed oral anticoagulants (OACs), including warfarin or direct oral anticoagulants (DOACs), within six months prior to ablation.

### Outcomes

We investigated the incidence of new-onset adverse events related to AF catheter ablation during the one-month follow-up (procedure-related events), as well as adverse events associated with AF, catheter ablation, or anticoagulation during the two-year follow-up period (long-term events). A new-onset event was defined as the first-ever recorded ICD-10 diagnosis code for the event of interest in the hospital care register, occurring during the index ablation procedure or within the one-month and two-year follow-up periods. An event was classified as new-onset only if the diagnosis of the event was not recorded in any available registries before the ablation.

The studied adverse events included diagnoses of ischaemic stroke or transient ischaemic attack (IS/TIA), bleeding, heart failure, myocardial infarction, and all-cause mortality during two-year follow-up. Additionally, pericardial complications (pericarditis, hemopericardium, or the need for pericardiocentesis or surgical pericardiotomy), pneumothorax, and haemothorax were reviewed during one-month follow-up (Supplement S1). Moreover, we identified the incidence of the composite endpoint of adverse events, including new-onset IS/TIA, bleeding, heart failure, myocardial infarction, early pericardial complications and all-cause mortality, at the one-month and two-year follow-up. We also studied the incidence of IS/TIA in patients who discontinued OAC therapy after the ablation procedure.

### Study ethics

The study protocol was approved by the Ethics Committee of the Medical Faculty of Helsinki University, Helsinki, Finland (nr. 15/2017 and 15/2024) and received research permission from the Helsinki University Hospital (HUS/46/2018 and HUS/217/2024). Respective permissions were obtained from the Finnish register holders (KELA 138/522/2018; THL 2101/5.05.00/2018; Population Register Centre VRK/1291/2019-3; Statistics Finland TK-53-1713-18 / u1281; and Tax Register VH/874/07.01.03/2019). All Finnish inhabitants have a unique national identification number, and the patients’ individual data from Finnish nationwide population registers and regional laboratory databases were linked together, using this national identification code. The research group received individualized but pseudonymized and unidentifiable data which ensures full data protection of the patients. The study was conducted without any direct patient involvement or contact during any phase of the study. Therefore, no patient consent was needed according to the Finnish legislation. The study conforms to the Declaration of Helsinki as revised in 2024.

### Statistical analyses

Differences in categorical variables were compared using Chi-square test and continuous variables were compared using students *t*-test. The associations between baseline clinical characteristics and new‑onset adverse events were investigated using a Cox proportional hazards regression model. Adjusted hazard ratios (AHRs) and 95% confidence intervals (CIs) were calculated separately for each new-onset outcome. The proportional hazards assumption was evaluated using time‑dependent covariates. Because the proportional hazards assumption was violated for sex in the IS model, we modelled sex with a time‑varying coefficient by including a sex × time (days) interaction term in the Cox model. The assumption was satisfied for all other covariates. To minimize bias in the new-onset event analysis, patients with a prior event of interest were excluded. Age was included as a continuous variable, while the following covariates were entered as dichotomous variables: sex, a history of hypertension, diabetes, hyperlipidaemia, heart failure, coronary artery disease, IS/TIA, and bleeding. Covariates for adjustment were selected based on their known associations with AF and cardiovascular outcomes, as well as their common use in clinical risk stratification models. To mitigate multicollinearity, only unadjusted hazard ratios (HRs) were calculated and reported for the CHA₂DS₂‑VA score, which was modelled as a continuous variable and analysed separately. Additionally, we computed Kaplan-Meier curves to illustrate the incidence of composite endpoint of adverse events comparing patients aged < 70 years with those aged ≥ 70 years, as well as men with women. We tested several age cut-offs (70, 75, and 80 years) by dichotomizing age and evaluated their discriminatory power for the composite endpoint at two years using ROC curve analysis. The analysis showed that 70 years provided the best, though weak, discrimination (AUC = 0.542). A breakpoint analysis was performed to examine changes in the incidence of combined IS/TIA within a year after the ablation, and according to the identified breakpoint, incidence rates of IS/TIA of the early post-ablation period and the subsequent period were compared and assessed with Poisson regression. Adjusted incidence rate ratios for the first new‑onset IS/TIA event were calculated using a model that included age, sex, and comorbidities (hypertension, heart failure, dyslipidaemia, diabetes, and coronary artery disease). Patients were removed from the risk set after their first event. Moreover, we evaluated the incidence of IS/TIA after the discontinuation of anticoagulant therapy within the two-year follow-up after ablation, defining discontinuation as 120 days after the last pharmacy purchase of OACs during the follow-up period. Statistical analyses were conducted using IBM SPSS Statistics software version 29.0 (SPSS, Inc., Chicago, Illinois, USA) and R version 4.0.5 (R Core Team). Breakpoint analysis was conducted using the segmented R package.

## Results

### Study population

A total of 3075 first-time AF ablations were performed during 2012–2016. The mean age of the patients was 57.8 years (range 21–83 years), the mean CHA_2_DS_2_-VA score was 1.4 and 29.4% of the patients were women (Table 1). Women were significantly older than men (60.8 years vs. 56.6 years, *p* < 0.001) and had a higher CHA_2_DS_2_-VA score (1.6 vs. 1.2, *p* < 0.001). The most common comorbidity was hypertension (60.8%), followed by hyperlipidaemia (27.8%) and coronary artery disease (15.5%). Moreover, 7.7% of the patients presented with a history of IS/TIA and 7.3% with a history of bleeding before catheter ablation. 80.0% of the patients had neither of studied endpoints (HF, IS/TIA, bleeding or myocardial infarction) before ablation.

**Table 1:** Clinical baseline characteristics

*n*	3075
Age, years	57.8 (9.7)
Range	21–83
Age ≥ 70 years	265 (8.6)
Female sex	905 (29.4)
Medication	
Warfarin	2124 (69.1)
DOAC	275 (8.9)
Anticoagulant NA	676 (22.0)
Betablockers	2634 (85.7)
Flecainide	1456 (47.3)
Amiodarone	578 (18.8)
Dronedarone	310 (10.1)
Sotalol	121 (3.9)
Digoxin	134 (4.4)
Comorbidities	
Hypertension	1871 (60.8)
Diabetes	214 (7.0)
Hyperlipidaemia	855 (27.8)
Heart failure	198 (6.4)
Coronary artery disease	478 (15.5)
IS or TIA	236 (7.7)
Previous bleeding	223 (7.3)
CHA_2_DS_2_-VA	1.4 (1.2)

Values denote n (%) or means (standard deviation). Abbreviations: *DOAC* direct oral anticoagulant, *IS* ischaemic stroke *NA* not available, CHA_2_DS_2_-VA score, congestive heart failure (1 point), hypertension (1 point), age ≥ 75 years (2 points), diabetes (1 point), history of stroke or TIA (2 points), vascular disease (1 point), age 65–74 years (1 point), *TIA* transient ischaemic attack

A total of 69.1% of the patients had documented purchase of warfarin and 8.9% of DOAC within six months prior to ablation. Among warfarin users the mean of the last measured International Normalized Ratio (INR) before the ablation was 2.3 (SD 0.5). Documentation on the reimbursed prescription or the use of anticoagulant was not available in 22.0% of the patients. Patients without identifiable OAC purchases had lower CHA_2_DS_2_-VA score in comparison to those with identifiable purchases (0.9 vs. 1.5, *p* < 0.001). The proportion of patients with identifiable DOAC purchases increased from 1.8% in 2012 to 24.5% in 2016, while the proportion of patients with unidentifiable OAC purchases increased from 7.2% in 2012 to 32.9% in 2016.

### Outcomes

At one-month follow-up 1.14% (35 patients), and at two-year follow-up 6.34% (195 patients) of the patients had experienced at least one new-onset adverse event or had died from any cause, thereby meeting the criteria for the composite endpoint (Table 2). There were no statistically significant differences or trends in the incidence of composite endpoint across different years during the study period. Patients who experienced adverse events during the two-year follow-up were older (mean age 61.1 years vs. 57.6 years, *p* < 0.001) and had higher CHA_2_DS_2_-VA- scores (1.7 vs. 1.3, *p* < 0.001) than those without events. All-cause mortality was low, 0.07% (2 patients) at one-month follow-up and 0.29% (9 patients) at two-year follow-up after the ablation. All-cause mortality was significantly higher in patients ≥ 70 years compared to those aged < 70 years (1.9% vs. 0.1%, *p* < 0.001).

**Table 2:** Incidence of new-onset adverse events within one month and two years after catheter ablation

	One month	One month to two years	Two years total
IS or TIA	10 (0.33)	46 (1.50)	56 (1.82)
IS	4 (0.13)	18 (0.59)	22 (0.72)
TIA	7 (0.23)	29 (0.94)	36 (1.17)
Bleeding	7 (0.23)	68 (2.21)	75 (2.44)
Heart failure	2 (0.07)	36 (1.17)	38 (1.24)
Myocardial infarction	2 (0.07)	15 (0.49)	17 (0.55)
Pericardial complication	12 (0.39)	NA*	12 (0.39)
Pneumo- or haemothorax	0 (0.00)	NA*	0 (0.00)
All-cause mortality	2 (0.07)	7 (0.23)	9 (0.29)
Any adverse event	35 (1.14)	160 (5.20)	195 (6.34)

Values denote n (%). Abbreviations: *IS* ischaemic stroke, *NA* not analysed, *TIA* transient ischaemic attack, *=only pericardial complications, pneumothorax, or haemothorax occurring during the one-month follow-up were included in the analysis and events beyond one month were not analysed

**Fig. 2:**
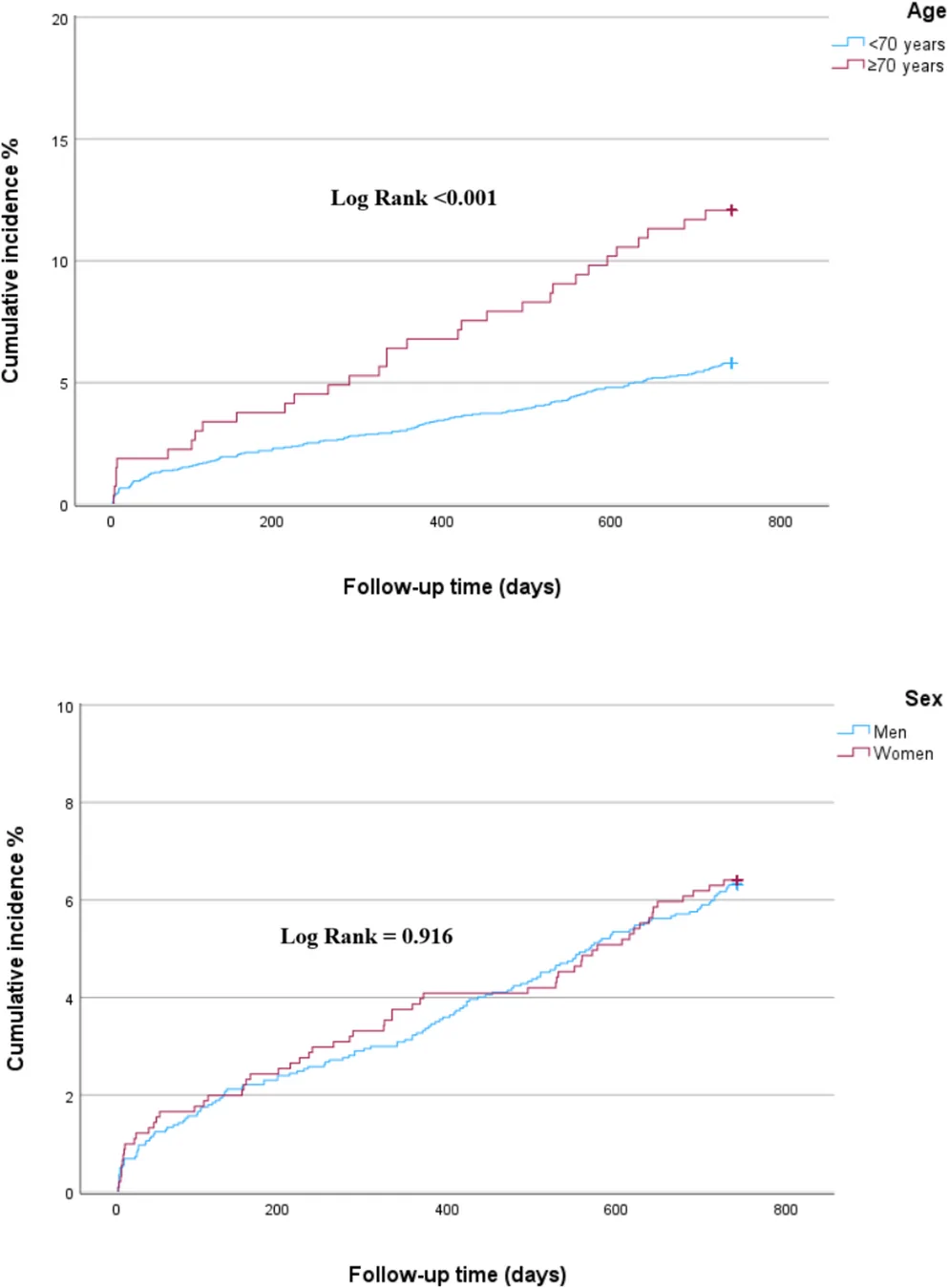
Cumulative incidence of the composite endpoint (new-onset ischaemic stroke, transient ischaemic attack, bleeding, heart failure, myocardial infarction, early pericardial complications, and all-cause mortality) in patients aged ≥ 70 years versus < 70 years (top panel), and in men versus women (bottom panel)

Kaplan–Meier subgroup analysis revealed a significantly higher incidence of the composite endpoint in patients aged ≥ 70 years compared to those aged < 70 years during the two-year follow-up (12.07% vs. 5.74%, *p* < 0.001, Fig. 2). Notably, the difference at the two-year follow-up remained statistically significant (*p* < 0.001) even when all-cause mortality was excluded from the analysis. However, no difference in the incidence of composite endpoint was observed between men and women (6.31% vs. 6.40%, *p* = 0.916; Fig. 2).

At one-month follow-up, the incidence of new-onset IS was 0.13% (4 patients) increasing to 0.72% (22 patients) at two-year follow-up. The incidence of new-onset IS/TIA at one month and two years were 0.33% (10 patients), and 1.82% (56 patients), respectively (Table 2). The incidence of new-onset bleeding was 0.23% (7 patients) at one month and 2.44% (75 patients) at two years. New-onset heart failure was observed in 0.07% of patients (2 patients) at one month, and in 1.24% of patients (38 patients) at two years. Although the use of DOACs increased during the study period, no statistically significant differences or trends in IS/TIA or bleeding were observed across the years 2012–2018.

**Fig. 3:**
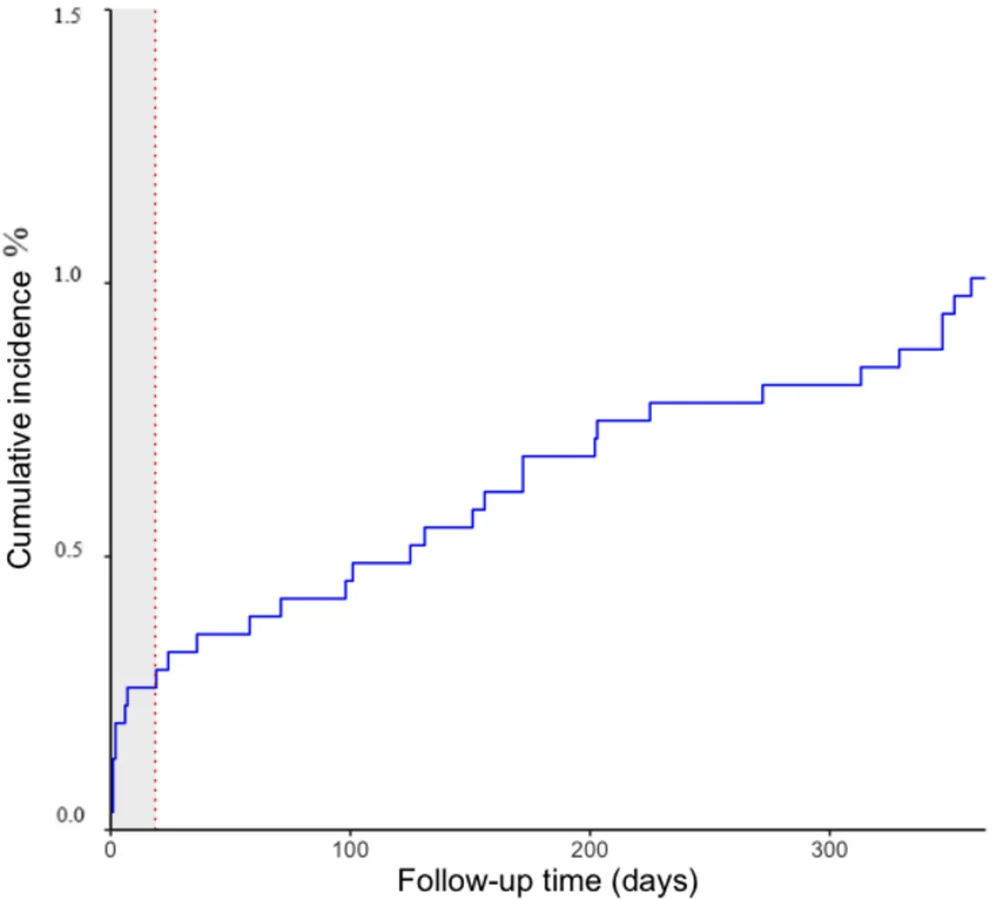
Cumulative incidence of new-onset ischaemic stroke or transient ischaemic attack (IS/TIA) following catheter ablation. The shaded area represents the period of peak incidence (first 19 days)

**Fig. 4:**
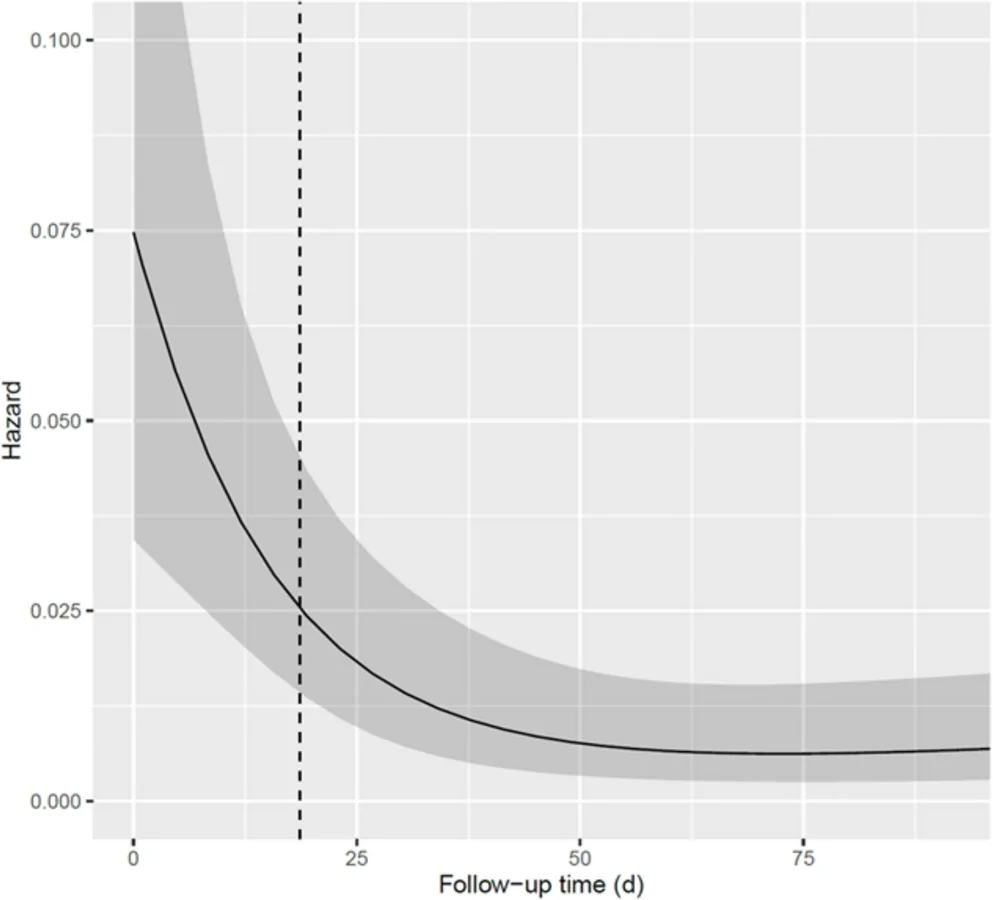
Hazard function for the incidence of ischaemic stroke or transient ischaemic attack (IS/TIA) after catheter ablation. The dotted line marks the breakpoint where the incidence rate begins to decline markedly

The breakpoint analysis identified a significant change in the incidence of IS/TIA at 19 days after the ablation (Figs. 3 and 4; Table 3). Both unadjusted and adjusted incidences of IS/TIA were 6.6 times higher before the breakpoint, that is, during the first 19 days after ablation (5.26 events per 100-patient years, 95% CI 2.27–10.36) compared to the subsequent follow-up period (0.79 events per 100-patient-years, 95% CI 0.50–1.19).

**Table 3:** Incidence rates of ischaemic stroke or transient ischaemic attack after catheter ablation

Days after ablation	*P*-years	Events, *n*	Incidence (per 100-*p*-years)	Unadjusted IRR (95% CI)	Adjusted IRR* (95% CI)
≤19 days	1.52	8	5.26 (2.27–10.36)	6.64 (2.97–14.85)	6.64 (2.97–14.84)
20–360 days	29.06	23	0.79 (0.50–1.19)	1.0 (Reference)	1.0 (Reference)

Abbreviations: *CI* confidence interval, *IRR* incidence rate ratio, *IS* ischaemic stroke, *TIA* transient ischaemic attack, *P-year *patient-year. * = Adjustment for age, sex, hypertension, heart failure, dyslipidaemia, diabetes, and coronary artery disease

Among warfarin users, the mean of the last measured INR before new‑onset IS/TIA was 2.2 (SD 0.7). There was no significant difference in the last measured INR between men and women (2.1 vs. 2.6, *p* = 0.103). IS/TIA rate after the discontinuation of OAC was 0.82 (95% CI 0.48–1.29) events per 100 patient-years. OAC therapy was not significantly associated with the occurrence of IS/TIA, with unadjusted and adjusted incidence rate ratios (95% CI) of 1.20 (0.69–2.11) and 1.15 (0.63–2.08), respectively. Among the 18 patients who experienced IS/TIA after discontinuing anticoagulation, the mean age was 58.6 years (range 44–72), 14 (77.8%) were men, and the mean CHA₂DS₂-VA score was 1.0. The incidence of IS/TIA per 100 patient-years after OAC discontinuation was 0.88, 0.44, and 1.59 for CHA₂DS₂-VA scores of 0, 1, and ≥ 2, respectively.

**Fig. 5:**
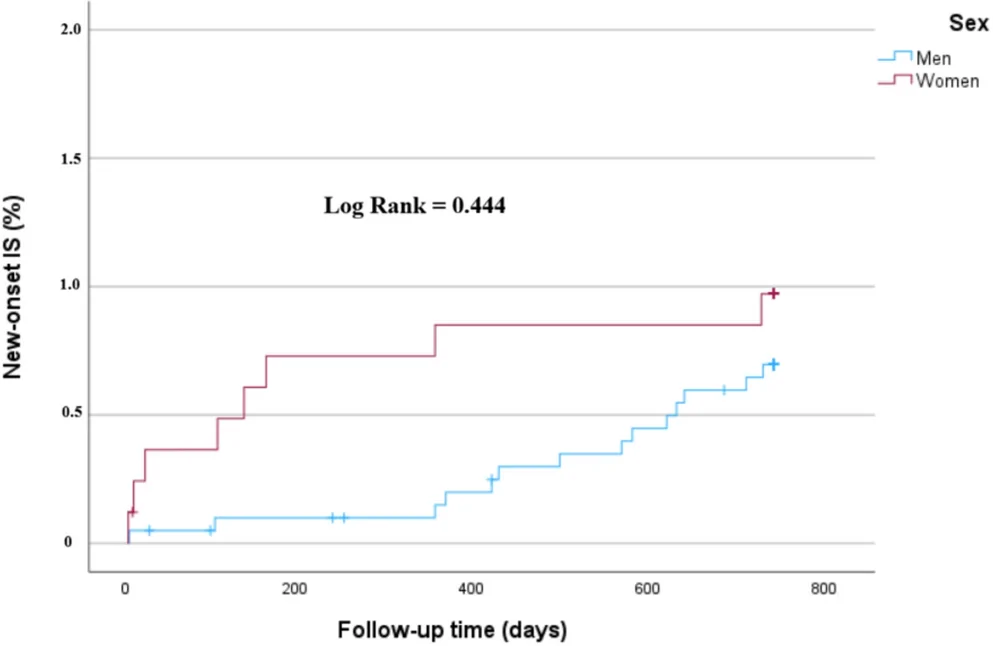
Cumulative incidence of new-onset ischaemic stroke in men versus women

In the adjusted Cox proportional hazards regression analysis, age was associated with new-onset bleeding (AHR 1.05, 95% CI 1.02–1.08, *p* = 0.002), new-onset heart failure (AHR 1.08 95% CI 1.03–1.13, *p* = 0.002) and all-cause mortality (AHR 1.16, 95% CI: 1.04–1.30, *p* = 0.007) during the two-year follow-up (Table 4). In addition, age and a history of IS/TIA were associated with the occurrence of new-onset heart failure, while a history of bleeding was associated with new-onset myocardial infarction. In the Cox proportional hazards model, none of the baseline clinical characteristics were predictors of combined IS/TIA. However, in the specific model for IS, women had a significantly higher baseline hazard than men (AHR at the time of ablation 6.40, 95% CI 1.19–34.36; *p* = 0.030) (Fig. 5, Supplementary material S2). The sex effect decreased over time, as indicated by a significant time interaction (HR per day 0.995, 95% CI 0.990–0.999; *p* = 0.023). The time‑specific hazard ratios were approximately 5.50 at 30 days, 2.59 at 180 days, and 1.02 at 365 days. Other covariates were not significant predictors of IS, and there were no significant predictors of TIA. Moreover, in the unadjusted analysis, the CHA₂DS₂‑VA score did not predict the occurrence of combined IS/TIA (HR 1.05, 95% CI 0.81–1.36, *p* = 0.730) or TIA alone (HR 0.75, 95% CI 0.52–1.08, *p* = 0.126). However, the CHA₂DS₂‑VA score was a significant predictor of IS during the two‑year follow‑up (HR 1.52, 95% CI 1.05–2.21, *p* = 0.027).

**Table 4:** Associations between pre-ablation clinical characteristics and new-onset adverse events during two-year follow-up after catheter ablation

Adverse event	Ischaemic stroke or TIA		Bleeding		Heart failure	
	AHR* (95% CI)	*p*-value	AHR* (95% CI)	*p*-value	AHR* (95% CI)	*p*-value
Baseline characteristics						
Age	1.01 (0.98–1.04)	0.405	1.05 (1.02–1.08)	0.002	1.08 (1.03–1.13)	0.002
Female sex	0.86 (0.47–1.58)	0.626	0.68 (0.40–1.16)	0.156	1.71 (0.90–3.28)	0.104
Coronary artery disease	1.11 (0.55–2.23)	0.768	1.25 (0.68–2.29)	0.469	1.30 (0.60–2.84)	0.507
Diabetes	0.87 (0.31–2.49)	0.800	0.97 (0.38–2.46)	0.940	1.27 (0.44–3.67)	0.663
Heart failure	0.84 (0.26–2.70)	0.772	1.94 (0.96–3.91)	0.064	NA	NA
Hyperlipidaemia	1.71 (0.94–3.12)	0.082	0.59 (0.33–1.05)	0.072	1.01 (0.50–2.01)	0.987
Hypertension	0.77 (0.44–1.34)	0.352	1.06 (0.65–1.72)	0.819	2.41 (0.98–5.92)	0.056
IS or TIA	NA	NA	1.46 (0.70–3.07)	0.318	2.73 (1.23–6.03)	0.013
Bleeding	0.72 (0.23–2.35)	0.594	NA	NA	0.22 (0.03–1.58)	0.131

Adverse event	Myocardial infarction		All-cause mortality			
	AHR* (95% CI)	*p*-value	AHR* (95% CI)	*p*-value		
Baseline characteristics						
Age	1.04 (0.98–1.11)	0.217	1.16 (1.04–1.30)	0.007		
Female sex	0.90 (0.31–2.60)	0.840	0.44 (0.09–2.15)	0.307		
Coronary artery disease	2.28 (0.77–6.75)	0.136	1.34 (0.31–5.87)	0.689		
Diabetes	1.13 (0.24–5.29)	0.881	2.53 (0.47–13.70)	0.282		
Heart failure	1.83 (0.41–8.09)	0.426	1.37 (0.16–11.48)	0.773		
Hyperlipidaemia	1.14 (0.38–3.39)	0.819	1.38 (0.33–5.83)	0.663		
Hypertension	0.78 (0.27–2.27)	0.652	1.24 (0.24–6.39)	0.799		
IS or TIA	0.57 (0.07–4.34)	0.587	2.65 (0.53–13.17)	0.235		
Bleeding	4.35 (1.48–12.83)	0.008	0.73 (0.09–6.26)	0.777		

Abbreviations: *AHR* adjusted hazard ratio, *CI* confidence interval, *IS* ischaemic stroke, *NA* not analysed, *TIA *transient ischaemic attack. * = adjustment for age, sex, coronary artery disease, diabetes, heart failure, hyperlipidaemia, hypertension, ischaemic stroke or transient ischaemic attack and previous bleeding

## Discussion

In this nationwide cohort study of all first-time AF catheter ablations performed between 2012 and 2016, catheter ablation was found to be a relatively safe procedure in terms of short-term complications. However, 6.34% of the patients experienced at least one new-onset adverse event during two-year follow-up. Older age was associated with all-cause mortality, new-onset heart failure, bleeding, and a higher incidence of the composite endpoint of adverse events at the two-year follow-up. Female sex was associated with higher baseline hazard for IS at the time of ablation, but the sex-related difference attenuated during the follow-up. Moreover, CHA₂DS₂‑VA score was a significant predictor of IS during the two‑year follow‑up.

Catheter ablation is an invasive and complex procedure that requires vascular access with large introducers, transseptal puncture, and the creation of ablation lines using radiofrequency energy, cryoablation, or, more recently, pulsed field ablation. Considering this, the incidence of procedure-related complications was reasonably low. At the one-month follow-up, 1.14% of patients experienced new-onset adverse events, and notably, the incidence of IS/TIA (0.33%) and all-cause mortality (0.07%) remained low. Thus, in our study the incidence of complications was somewhat lower than in previous reports, which have documented in-hospital complication rates ranging from 2.5% to 8.0%. In addition, mortality (0.07%) in our study was close to the lowest end of the previously reported range (0.05% to 0.45%) [23–25]. However, the event rates in our study should be interpreted in light of important differences from prior studies. Our cohort was relatively young (mean age 57.8 years) and had a low comorbidity burden (mean CHA₂DS₂‑VA score 1.4), and the analysis was restricted to first‑ever events only. These factors likely contributed to the lower incidence of adverse events observed compared with prior publications. Wu et al. reported a temporal increase in procedure-related complications between 2015 and 2017, driven by pericardial complications. In contrast, we found no temporal change in the rate of complications during the period of interest, 2012–2018.

During the two-year follow-up new-onset adverse events were primarily driven by bleeding (2.44%), IS/TIA (1.82%) and heart failure (1.24%). However, mortality remained low at 0.29%. In the CABANA trial, a randomized study comparing catheter ablation and drug therapy among patients with AF, the four-year mortality rate in the catheter ablation group was 4.7% [20]. Correspondingly, a registry study from Sweden reported all-cause mortality of 2.1% during 4.5-year follow-up [26]. However, direct comparisons between these studies and our study should be interpreted with caution due to potential differences in patient selection, as well as variations in study populations and follow-up durations.

Catheter ablation is increasingly utilized in elderly populations [7, 27, 28]. In our study, the mean patient age was modest, 57.8 years. However, older patients were more susceptible to adverse events, particularly all-cause mortality, bleeding, heart failure, and the composite endpoint of adverse events. Our findings agree with most previous studies [12–15, 20], but not all [11]. Thus, our study supports the current recommendations that decisions regarding ablation should be based on a careful evaluation of potential quality-of-life benefits weighed against the risks of serious adverse events, particularly in elderly patients.

Several studies have reported that women have a higher risk of procedural complications after AF ablation and this may be one of the reasons that women are less likely to undergo AF ablation than men [17, 29–33]. Accordingly, in our cohort, most patients undergoing AF ablation were men. Women had a higher risk of IS early after ablation. However, they were also older and had a greater comorbidity burden and higher CHA₂DS₂‑VA score. This observed sex‑related disparity in ablation populations does not appear to be explained solely by concerns about an increased risk of complications. Still, it is possible that women were referred for ablation more selectively than men, given the common belief that they are at higher risk of procedural complications.

The risk of IS/TIA remained elevated for approximately three weeks (19 days) after the ablation procedure, which is consistent with previous studies [23, 34, 35]. Potential mechanisms of thromboembolic complications after ablation include thrombus formation on sheaths or catheters in the left atrium, catheter-tip char, endothelial injury and thrombus formation along ablation lines, dislodgment of a pre-existing left-atrial thrombus, and possible electrical cardioversion performed during the procedure [23]. However, a Swedish observational, propensity score–matched study reported that catheter ablation was associated with lower all-cause mortality and showed a trend toward reduced stroke risk compared to medical management in patients with AF during long-term follow-up [26]. On the other hand, the CABANA trial, found no significant difference in the incidence of IS between the catheter ablation and the drug therapy groups over a median follow-up of four years [20]. In our study, the risk of IS/TIA following ablation was 1.82% in the overall cohort during the two-year follow-up period and 0.8% per year after discontinuation of anticoagulation. Two recent randomized studies reported low IS rates of 0.3% during two- and three-year follow-up periods in patients receiving aspirin after discontinuation of OAC [36, 37]. However, direct comparisons between our study and these randomized trials should be avoided, as anticoagulation in those trials was discontinued only in patients without documented AF after ablation, whereas information on AF recurrence was unavailable in our study. The recent study by Kim et al. found that the risk of IS/TIA was not associated with CHA₂DS₂-VA scores. In our study, The CHA₂DS₂-VA score was a significant predictor of new-onset IS after ablation but could not predict IS/TIA or TIA alone. The CHA₂DS₂-VASc and CHA₂DS₂-VA scores were originally developed and validated in unanticoagulated AF populations, rather than specifically in patients undergoing ablation [38–40]. Therefore, these scores may not be appropriate for identifying patients at elevated thromboembolic risk following ablation, and also alternative risk stratification approaches may be required. On the other hand, because IS is the most feared thromboembolic complication after AF ablation, it is reassuring that the CHA₂DS₂‑VA score was able to predict it in our nationwide cohort.

Bleeding was the most frequent adverse event during the two-year follow-up period (2.44%). The incidence of bleeding complications after AF catheter ablation varies across studies, with current estimates of the incidence of vascular complications ranging from 1 to 4% [23]. Consistent with our finding of a 0.23% bleeding incidence during the one-month follow-up, a large German analysis across four high-volume tertiary centers reported a similarly low 0.2% incidence of in-hospital major vascular access complications, including bleeding, among patients undergoing AF ablation [41]. Additionally, in our cohort, older age was associated with bleeding complications during two-year follow-up. Notably, most patients were treated with warfarin, although the use of DOAC increased over the study period. This should be considered when assessing the generalizability of our findings, as most patients undergoing AF ablation today are treated with DOACs. However, despite the increasing use of DOACs over the study period, no significant temporal changes in the incidence of bleeding were observed. Because study endpoints were identified using ICD‑10 diagnosis codes and detailed clinical records were unavailable, we could not determine whether all bleeding events met major bleeding criteria. Nevertheless, these new‑onset bleeding events were likely clinically relevant, as they represented new hospital‑recorded diagnoses without prior documentation.

Associations and predisposing factors for heart failure following AF catheter ablation have been widely studied. CASTLE-AF trial found that catheter ablation significantly reduced hospitalizations for worsening heart failure and all-cause mortality in patients with AF and heart failure with reduced ejection fraction [9]. Moreover, The CASTLE-HTx study compared catheter ablation with medical therapy in patients with symptomatic AF and end-stage heart failure, finding that ablation combined with guideline-directed therapy reduced the risk of a composite outcome of all-cause death, left ventricular assist device implantation, or urgent heart transplantation compared with medical therapy alone [42]. Åkerström et al. reported greater clinical benefit from catheter ablation in patients with a history of heart failure, and a lower incidence of new-onset heart failure among those who underwent ablation [26]. In our cohort, the incidence of new-onset heart failure during the two-year follow-up was low (1.24%). Older age, a history of hypertension, and prior IS/TIA were significant predictors of new-onset heart failure.

## Strengths and limitations

A major strength of this study is the use of a comprehensive nationwide dataset that includes all patients with AF and AFL in Finland. This dataset covers all first-time AF catheter ablation procedures performed between 2012 and 2016, along with complete healthcare contact records for two years following the ablation. This provided a unique opportunity to evaluate the outcomes of patients undergoing AF catheter ablation during long-term follow-up in an unselected, real-world nationwide cohort. The registers used in this study have been shown to be highly reliable, particularly in identifying cardiovascular diseases [43], [https://www.worldbank.org/en/programs/statistical-performance-indicators]. Because most ICD‑10 codes do not distinguish between new and recurrent events, and we did not have access to patients’ medical records, we limited our analyses to new‑onset (first‑ever) adverse events to estimate the incidence of the outcomes of interest as accurately as possible. Importantly, 80.0% of patients had not experienced any of the studied endpoints (HF, IS/TIA, bleeding, or myocardial infarction) before ablation, and the cohort generally had low CHA₂DS₂‑VA scores. Thus, we believe that our study provides a valuable description of the risk of adverse events in a relatively healthy, real‑world population undergoing first‑time AF ablation.

On the other hand, we also acknowledge some limitations, most of which are typical of register-based retrospective cohort studies. Although restricting outcomes to first‑ever diagnoses strengthens methodological clarity, it also introduces clinical limitations, as the data on recurrent adverse events were not available. Moreover, there is a possibility of inaccuracies in data recording, such as underreporting of complications via ICD-10 codes. For instance, specific ICD‑10 codes do not exist for certain rare but serious AF ablation complications, such as atrioesophageal fistula and phrenic nerve palsy. Consequently, these events could not be assessed in our study, and this should be taken into account when interpreting our observed complication rates. In addition, some essential data related to AF catheter ablation were unavailable, including the ablation method used, the number of procedures performed per physician or centre, AF-related symptoms, AF recurrences, AF burden, body mass index, and echocardiographic data.

A substantial proportion of patients had missing data on the use of anticoagulation. In Finland, AF ablations are centralized to university hospitals and large central hospitals, and current guidelines are followed closely. All patients referred for AF ablation in Finland are routinely anticoagulated, including those with low or intermediate CHA₂DS₂‑VA scores (scores 0–1). Our dataset includes only drug purchases reimbursed by the Social Insurance Institute, and at that time DOACs were not reimbursed for low‑ or intermediate‑risk patients undergoing AF ablation. Most patients without identifiable OAC purchases indeed had low or intermediate CHA₂DS₂‑VA scores, meaning they were required to purchase DOACs without reimbursement. Consequently, these purchases were not recorded in the National Reimbursement Register. During the study period, the use of warfarin declined and the use of DOACs increased, and the proportion of non‑identifiable OAC purchases rose accordingly. The most likely explanation is that the patients without recorded OAC use purchased DOACs without reimbursement, and these purchases were therefore not captured in our data. This limitation should be taken into account when interpreting our analyses on OAC discontinuation.

In the recent years, AF ablation has progressed rapidly, particularly with pulsed field ablation demonstrating a safety profile comparable to, or potentially more favourable than, thermal techniques [44–46]. These advances may have altered the landscape compared to the findings of our study. On the other hand, pulmonary vein isolation, using either radiofrequency or cryoballoon techniques, remains the most commonly employed method for AF catheter ablation [47].

## Conclusion

In conclusion, this comprehensive nationwide cohort study, including all first-time catheter ablations for AF in Finland between 2012 and 2016, demonstrated that the incidence of early new-onset adverse events following ablation was relatively low. Older age was associated with increased mortality and a higher incidence of bleeding, heart failure, and a composite endpoint of adverse events during the two-year follow-up. The incidence of IS/TIA was highest within approximately three weeks post-ablation but remained low thereafter. Women presented with a higher risk of IS at the time of ablation, but female sex was not associated with other adverse events.

## Supplementary information

Below is the link to the electronic supplementary material.

Supplementary Material 1

## Data Availability

Because of the sensitive nature of the data collected for this study, requests to access the dataset from qualified researchers trained in human subject confidentiality protocols may be sent to the Finnish national register holders (KELA, Finnish Institute for Health and Welfare, Population Register Center and Tax Register) through Findata ( [https://findata.fi/en/](https:/findata.fi/en) ).

## Abbreviations

AF: Atrial Fibrillation
AFL: Atrial Flutter
DOAC: Direct Oral Anticoagulant
IS: Ischaemic Stroke
PVI: Pulmonary Vein Isolation
TIA: Transient Ischaemic Attack

## Declarations

### Registration number of clinical studies

Clinical Trials Identifier:NCT04645537; ENCePP Identifier: EUPAS29845
