# Supplementary material for: Adverse events after catheter ablation of atrial fibrillation: a comprehensive nationwide cohort study
Source: J Interv Card Electrophysiol. 2026 Apr 21;69(8):2359–70. doi: 10.1007/s10840-026-02328-4 (PMC13740091; doi:10.1007/s10840-026-02328-4)
Supplement: Supplementary file 1 [file 10840_2026_2328_MOESM1_ESM.docx]

**Adverse events after catheter ablation of atrial fibrillation: a comprehensive nationwide cohort study**

**Supplementary material**

**Supplementary table S1.** Definitions of the comorbidities, procedures and outcomes according to ICD-10 and NCSP (Nordic classification of surgical procedures)

| **Bleeding** | D50.0, D62, D68.3, I60, I61, I62, I85.0, I86.4, J94.2, K22.1, K22.3, K22.6, K25.0, K25.2, K25.4, K25.6, K26.0, K26.2, K26.4, K26.6, K27.0, K27.2, K27.4, K27.6, K28.0, K28.2, K28.4, K28.6, K29.0, K62.5, K63.1, K63.3, K92.0, K92.1, K92.2, N02, R04, R31, R58, S06.3, S06.4, S06.5, S06.6, S06.8 |
| --- | --- |
| **Hypertension** | I10, I11, I12, I13, I14, I15 |
| **Hyperlipidaemia** | E78 |
| **Diabetes** | E10, E11, E12, E13, E14 |
| **Ischaemic stroke or TIA** | I63, I64, G45 |
| **Ischaemic stroke** | I63, I64 |
| **TIA** | G45 |
| **Coronary artery disease** | I20, I21, I22, I23, I24, I25 |
| **Myocardial infarction** | I21, I22 |
| **Heart failure** | I50, I11.0, I13.0, I13.2 |
| **Hemopericardium** | I31.2, I31.3, I31.9, S26.0 |
| **Pericarditis** | I30.0, I30.8, I30.9 |
| **Pneumothorax** | J93.8, J93.9, S27.0 |
| **Pericardiocentesis or drainage** | TFE00, FEA |
| **Surgical pericardiotomy** | FEB, FEC |

**Supplementary table S2.** Adjusted hazard ratios between pre-ablation clinical characteristics and new-onset ischaemic stroke during two-year follow-up after catheter ablation.

| **Adverse event** | **Ischaemic stroke** | |
| --- | --- | --- |
| n | 22 |  |
|  | **AHR* (95% CI)** | **p-value** |
| **Baseline characteristics** |  |  |
| Age (per year) | 1.04 (0.98-1.10) | 0.195 |
| Female sex (#) | **6.40 (1.19-34.36)** | **0.030** |
| Female sex x Time (└─) | 0.995 (0.990-0.999) | **0.023** |
| Coronary artery disease | 1.46 (0.54-3.96) | 0.454 |
| Diabetes | 1.43 (0.40-5.09) | 0.580 |
| Heart failure | 1.38 (0.32-5.94) | 0.664 |
| Hyperlipidaemia | 1.61 (0.63-4.09) | 0.316 |
| Hypertension | 1.29 (0.48-3.45) | 0.611 |
| Bleeding | 0.49 (0.06-3.67) | 0.484 |
| Abbreviations: AHR, adjusted hazard ratio; CI, confidence interval; IS, ischaemic stroke; NA, not analysed; TIA, transient ischaemic attack. * = adjustment for age, sex, coronary artery disease, diabetes, heart failure, hyperlipidaemia, hypertension, and previous bleeding. # = adjusted hazard ratio at the time of ablation. └─ =Time‑varying coefficient (per day). | | |

**Supplementary table S3.** Adjusted hazard ratios between pre-ablation clinical characteristics and new-onset transient ischaemic attack during two-year follow-up after catheter ablation.

| **Adverse event** | **Transient ischaemic attack** | |
| --- | --- | --- |
| n | 36 |  |
|  | **AHR* (95% CI)** | **p-value** |
| **Baseline characteristics** |  |  |
| Age (per year) | 1.00 (0.97-1.04) | 0.985 |
| Female sex | 0.74 (0.33-1.66) | 0.463 |
| Coronary artery disease | 0.81 (0.30-2.17) | 0.670 |
| Diabetes | 0.38 (0.05-2.88) | 0.351 |
| Heart failure | 0.46 (0.06-3.33) | 0.439 |
| Hyperlipidaemia | 1.91 (0.89-4.09) | 0.098 |
| Hypertension | 0.58 (0.29-1.16) | 0.121 |
| Bleeding | 0.88 (0.21-3.68) | 0.859 |
| Abbreviations: AHR, adjusted hazard ratio; CI, confidence interval; IS, ischaemic stroke; NA, not analysed; TIA, transient ischaemic attack. * = adjustment for age, sex, coronary artery disease, diabetes, heart failure, hyperlipidaemia, hypertension, and previous bleeding. | | |
